# Involvement of Versatile Bacteria Belonging to the Genus *Arthrobacter* in Milk and Dairy Products

**DOI:** 10.3390/foods12061270

**Published:** 2023-03-16

**Authors:** Nuthathai Sutthiwong, Supaporn Lekavat, Laurent Dufossé

**Affiliations:** 1 Expert Centre of Innovative Health Food (InnoFood), Thailand Institute of Scientific and Technological Research, Technopolis 35 Mu 3, Klong Ha, Klong Luang, Pathum Thani 12120, Thailand; 2 Chimie et Biotechnologie des Produits Naturels (CHEMBIOPRO), ESIROI Département Agroalimentaire, Université de La Réunion, Parc Technologique, 2 rue Joseph Wetzell, F-97490 Sainte-Clotilde, Ile de La Réunion, France

**Keywords:** *Arthrobacter*, application, milk, dairy products

## Abstract

Milk is naturally a rich source of many essential nutrients; therefore, it is quite a suitable medium for bacterial growth and serves as a reservoir for bacterial contamination. The genus *Arthrobacter* is a food-related bacterial group commonly present as a contaminant in milk and dairy products as primary and secondary microflora. *Arthrobacter* bacteria frequently demonstrate the nutritional versatility to degrade different compounds even in extreme environments. As a result of their metabolic diversity, *Arthrobacter* species have long been of interest to scientists for application in various industry and biotechnology sectors. In the dairy industry, strains from the *Arthrobacter* genus are part of the microflora of raw milk known as an indicator of hygiene quality. Although they cause spoilage, they are also regarded as important strains responsible for producing fermented milk products, especially cheeses. Several *Arthrobacter* spp. have reported their significance in the development of cheese color and flavor. Furthermore, based on the data obtained from previous studies about its thermostability, and thermoacidophilic and thermoresistant properties, the genus *Arthrobacter* promisingly provides advantages for use as a potential producer of β-galactosidases to fulfill commercial requirements as its enzymes allow dairy products to be treated under mild conditions. In light of these beneficial aspects derived from *Arthrobacter* spp. including pigmentation, flavor formation, and enzyme production, this bacterial genus is potentially important for the dairy industry.

## 1. Introduction

Milk is a rich source of protein and a whole range of nutrients necessary for growth, including fats, carbohydrates, vitamins, minerals, and essential amino acids. Related to its proteins and peptides, milk also provides several physiological functions such as immunoglobulins, enzymes, growth factors, hormones, and antibacterial agents [1]. All milk obtained from a variety of animal sources has a nearly neutral pH and a high-water activity [2]. Due to the requirement of appropriate nutrition for health benefits and body growth and development as well as body maintenance and protection from diseases, milk becomes a vital source of nutrition for humans. With the rapid rise in population growth, increasing income, urbanization, and changes in consumption habits, in addition to the normal consumption of milk as fluid, global demand for milk-derived products is continuing to grow.

In addition to being an excellent source of human nutrition, milk has been referred to as a great environment for the survival and growth of a wide range of microorganisms because of its near-neutral pH, high water content, and complex biochemical compositions. As an agricultural product, milk is collected from domesticated animals, particularly cows. The number and types of microorganisms exhibited in milk are affected by the seasons, farm hygiene, feed, and efficiency of storage. The microorganism found in milk can be categorized into three groups depending on their involvements, namely, pathogenic, spoilage, and fermented-food-derived bacterial strains [3,4]. Some nutrients contained in milk can be used directly by all microorganisms, while others are provided following the types of microorganisms presented such as lactose, which is not utilized by many bacteria, and large molecules—e.g., proteins and lipids, which must be broken down by enzymes before allowing sustained microbial growth [5]. Changes in biochemical activities in microbial communities during nutrient utilization led to the different characteristics of milk depending on the period of these activities; in this way, microbes beneficially associate with milk and dairy product technology. With accumulating knowledge about the functionality of microorganisms, it became less complicated to apply them in technological processes.

Nowadays, microorganisms are widely used in the food industry to produce various types of products due to their desirable properties. In the dairy industry, the utilization of microorganisms has been of increasing interest since it is an important part of quality control as well as quality development. Due to the up-to-date technologies developed over the past few decades, a variety of dairy products are available. In addition to lactic acid bacteria that are commonly present in milk, many psychrotrophic bacteria are also a major component frequently consisting of *Actinobacteria*. The particular composition of milk microbiota directly influences the development of dairy products. Microorganisms can trigger the fermentation of milk causing the production of lactate as well as the different effects on the organoleptic properties of fermented resultants [6].

*Arthrobacter* is a genus of bacteria that belongs to the ecologically and industrially important class *Actinobacteria* of the *Micrococaceae* family. This genus is an outstanding group of bacteria isolated from various sources and has been found to produce a great variety of pigment hues [7]. *Arthrobacter* strains are usually present as a contaminant in milk and play different roles in inducing diverse aspects of contaminated milk [8]. The genus *Arthrobacter* bacteria are widely distributed in nature even in various extreme environments as they are heterotrophic and do not require very attentive growth conditions. Bacteria of this genus are Gram-positive, catalase-positive, obligated aerobic, and non-spore-forming bacteria, with an optimum growth below 30 °C; moreover, several strains isolated from glacial environments are known as psychrophiles with an ability to grow well under below 0 °C [9,10]. At present, approximately 80 species of this genus have been described in the taxonomy [11]. The taxonomy of the genus *Arthrobacter* and related genera has been reclassified several times; therefore, some strains were originally recognized as belonging to the *Arthrobacter* genus but have been presently identified as other genera, and vice versa.

Certain well-known *Arthrobacter* species enclose a variety of desirable members of technological relevance to dairy products such as *Arthrobacter arilaitensis* and *Arthrobacter bergeri* (recently named *Glutamicibacter arilaitensis* and *Glutamicibacter bergeri*, respectively—taxonomy always evolves and we choose to keep the name *Arthrobacter arilaitensis* and *Arthrobacter bergeri* in this publication, in agreement with previous articles), which respond to color development in smear-ripened cheeses [12]. Regarding the profitable impacts of microorganisms on the dairy industry, this review aims to focus on the association of versatile bacteria belonging to the genus *Arthrobacter* with their significance in the applications to commercial milk and dairy products. The involvement of *Arthrobacter* in the color and flavor of cheeses and the potential of being novel sources for the commercial production of an industrially important enzyme “β-galactosidase” or other enzymes used in food processing, as well as the feasibility to utilize *Arthrobacter* spp. in the production of functional dairy products are mentioned in this review.

## 2. Occurrence of *Arthrobacter* spp. in Raw Milk and Dairy Products

Microorganisms in raw milk are varied and derived from several sources of contamination. When synthesizing within the mammary gland of healthy animals, milk is particularly expected to be sterile and safe for human consumption. Nevertheless, in addition to this stage, microbial contamination in raw milk possibly arises from diverse origins including the external udder surface, feed, farm environment, geographic location, and the equipment used for milk handling and storage. Hence, the occurrence of microorganisms in milk is rather influenced by various factors and their combinations, and it is often subsequently a cause of microbial contamination in dairy products in addition to several microorganisms that can emerge from the processing and post-processing lines [13,14].

Members of the genus *Arthrobacter* are commonly present in raw milk as a primary microflora and are believed to enter from the milking equipment as well as the teat surface [15,16]. Outdoor environments such as air in the byre and milking parlor and hay were also regarded as the sources of *Arthrobacter* spp. [17,18,19]. Certain methods such as culture-dependent and molecular approaches were applied as tools to classify *Arthrobacter* species that existed in raw milk [20,21]. Despite the microbial composition of milk having been investigated by clarifying several groups of microorganisms, few studies reported the presence of *Arthrobacter* spp. in raw milk; furthermore, the detail of species diversity of these bacteria has been much less observed. In recent studies, bacteria in the genus *Arthrobacter* were found to be one of the microbial communities in raw milk, accounting for approximately 1–2% of the total [21,22].

*Arthrobacter* spp. are rarely found predominantly in milk, although organic sources are not a restraining factor for the growth of *Arthrobacter* strains; instead, the competition of other microorganisms and the change in pH by the production of lactic acid or other organic acids can prevent the number of bacteria of this genus from growing. Consequently, *Arthrobacter* species isolated from raw milk and dairy products normally appeared as co-cultures with other microorganism strains, such as coryneform, micrococci, staphylococci, and yeasts [8]. Several *Arthrobacter* species, such as *Arthrobacter aurescens*, *A. arilaitensis*, *A. bergerei*, *Arthrobacter globiformis*, *Arthrobacter citreus*, *Arthrobacter nicotanae*, and *Arthrobacter bussei*, were identified in food, especially in raw milk and cheese. Since *Arthrobacter* species are aerobes, they grow mainly on the surface of food and feasibly cause deterioration of food, ripening, and smearing; thus, these strains are assumedly one of the main microbiota on cheese rind and contribute to differences in organoleptic characteristics such as flavor and color [17,21].

## 3. Significance of *Arthrobacter* spp. in the Dairy Industry 

Bacteria belonging to the genus *Arthrobacter* frequently demonstrate the nutritional versatility to degrade both natural and synthetic organic compounds as well as exhibit extreme resistance to dryness and starvation [23]. According to their metabolic diversity, *Arthrobacter* species have long been a topic of interest to scientists for application in various industry and biotechnology sectors. In the dairy industry, *Arthrobacter* strains are part of the microflora of raw milk known as an indicator of hygiene quality. They cause spoilage; however, they are also regarded as one of the important strains responsible for producing fermented milk products, especially cheeses.

### 3.1. Association of Arthrobacter spp. with Cheese Production

Cheese can be produced from raw or pasteurized milk. Certain cheese makers apply a sub-pasteurization heat treatment called thermization with the range of 62–65 °C for 10–20 s. This technique greatly reduces the number of spoilage bacteria with minimum collateral heat damage to milk components in order to prevent excessive gas openings and off-flavors in cheeses [24]. Nevertheless, cheese is still considered a complex microbial ecosystem, consisting of various species of microorganisms that vary in terms of number and composition including both yeasts and bacteria since they have not originated only from milk but also from starter cultures and cheese-making environments. The microbiota of cheese is a significant factor in the typicity and quality of cheese as the microbial interactions among microorganisms in raw milk as well as those introduced during production will influence the characteristics of cheese products. The change in biochemistry due to the interactions of microorganisms will be an indicator of the organoleptic properties, e.g., color, flavor, and texture, that are naturally developed during the cheese-making processes, especially ripening [25,26]. The rind of cheeses, particularly smear-ripened cheeses, is an obvious example that occurs depending on biochemical changes throughout cheese production. 

The presence of bacteria of the genus *Arthrobacter* in cheeses, especially smear-ripened and mold surface-ripened cheeses, has previously been reported. *Arthrobacter* species have been identified as one of the important bacteria among a diversity of microorganisms in smear-ripened cheeses due to their occurrence at different stages of the ripening process [27,28]. Theoretically, surface ripening begins with the growth of yeasts, depending on the cheese variety, mainly *Debaryomyces hansenii*, *Kluyveromyces marxianus*, *Yarrowia lipolytica*, and the yeast-like mold *Geotrichum candidum*, which colonize the cheese surface and metabolize lactic acid and provide growth factors useful to bacteria during the initial period of ripening [18,19,22]. The utilization of lactic acid by yeasts progressively leads to the deacidification of the cheese surface, allowing the growth of acid-sensitive bacteria and eventually covering the entire cheese surface. Several bacteria of the genus *Arthrobacter* were found to be dominant strains at the end of the ripening of smear-ripened cheeses [29].

*Arthrobacter* spp., together with other bacterial strains and yeasts, are the major microorganisms that occurred in cheeses from different origins. From several kinds of Austrian cheeses manufactured by different plants, numerous isolates were preliminarily identified *as A. globiformis* [23]. Several *Arthrobacter* strains were also previously found on the surfaces of different cheese varieties—e.g., *A. aurescens*, *A. citreus*, *A. globifomis*, *Arthrobacter protophormiae*, *Arthrobacter uratoxydans,* and *Arthrobacter variabilis*, whereas most *Arthrobacter* strains could not be identified as any known species [30]. In addition, some strains among *Arthrobacter* species namely *Arthrobacter rhombi* and *Arthrobacter sulfureus* were isolated from Limburger cheeses—smear-ripened semi-soft cheeses widely produced in Belgium, Germany, and Netherlands [31]. Although the occurrence of *Arthrobacter* spp. on the surface ripening of smear-ripened cheeses was often determined, only a few studies have taxonomically identified these isolates. With the development, improvement, and progress of technology during recent decades, *Arthrobacter* strains were additionally encountered. Using a modern technique—rDNA-based—in combination with a traditional method—cultivation—strains of *A. arilaitensis* have been reported to be one of the major bacterial species found at the surface of soft red-smear cheeses, e.g., French Livarot, Munster, Maroilles, and Reblochon [27,32]. In addition to *A. arilaitensis*, the presence of *A. bergerei* was also exhibited in these French cheeses [33]. Colonies of *Arthrobacter* strains having been found at different stages of ripening during cheese production, it is then assumed that *Arthrobacter* spp. is one of the major microorganisms contributing to the typical texture, flavor, and color properties of the final product [27,28,34].

#### 3.1.1. Implication of *Arthrobacter* spp. in Cheese Color

Similar to a large number of other products, the appearance of cheeses, especially smear-ripened cheeses, is a key element in a consumer’s purchasing decision due to it appears as a sign of quality. In this respect, the color, which is the result of pigment synthesis by complex surface microflora, is associated with some qualities of cheeses such as cleanliness, flavor, and maturity [35]. Irregular color characteristics of cheeses can raise economic loss to producers because it leads to consumers misunderstanding the quality of cheeses [36]. Smear-ripened cheeses, also called red-smear cheeses, are commonly characterized by hues of orange to the orange-reddish of microbial mat on the cheese rinds. Accordingly, color plays an important role that possibly affects consumer perception and acceptance.

In the past, *Brevibacterium linens* was considered to be the sole microorganism responsible for the color development at the surface of the cheeses because of its ability to produce orange carotenoids [37]. Other bacteria including cream-colored and yellow-pigmented coryneform were lately identified to be as significant as *B. linens* for the ripening of the cheese rind [25,30,37,38,39,40]. According to Bockelmann, 2002 [41], the color development of typical light-brown cheese such as Tilsit, Chaumes, Limburger, and Romadour was attributed to the interactions between yellow-pigmented *A. nicotianae* and other microorganisms while the orange-pigmented *B. linens* were found less significant. These findings confirmed the results of the earlier study by Eliskases-Lechner and Ginzinger [30]. *A. aurescens*, in co-culture with *Zymomonas mobilis*, was found to produce an abnormal yellow discoloration in yogurts and red-orange streaks on Italian cheeses [42]. One important strain among the genus *Arthrobacter* found in ripened cheeses is *A. arilaitensis* [12,27,39]. Colonies of *A. arilaitensis* commonly exhibit yellow colors and these bacteria have been found at different stages of ripening. It is then assumed that *A. arilaitensis* could be one of the microorganisms that would produce pigments on cheese rind, contributing to its characteristic overall color. [27,28,34]. The color is due essentially to carotenoids, in combination with other pigments, produced by the cheese microflora during ripening [43,44,45]. Based on UV–Visible (UV–Vis) spectroscopy, mass spectroscopy, and the elution order, the yellow pigments produced by the *A. arilaitensis* strains isolated from smear-ripened French cheeses were characterized as a group of C_50_ carotenoids consisting of eight forms, mainly decaprenoxanthin [46]. *Arthrobacter agilis* and *A. bussei* are also known as rare C_50_ carotenoid-producing bacterial strains [47]. Until now, approximately 1100 carotenoids from different organisms have been identified and classified based on the number of carbons in their carotene backbones [48]. Most of the natural carotenoids are subjected to the symmetric C_40_ backbone while a small number of C_30_ and C_50_ carotenoids have been classified [49,50].

Cheese is commonly aged isothermally at a low temperature depending on conditions optimized by the type of cheese, and the coloration of cheese is regularly developed during this process [51,52]. Low temperatures usually inhibit or stop microbial growth and proliferation; however, the genus *Arthrobacter*, which is known as a group of cold-adaptive microbes, would be involved in multiple mechanisms caused by low-temperature adaptation [53]. Carotenoids produced by *Arthrobacter* strains found in cheeses are presumably part of the cold adaptation mechanism of the strains. Carotenoids are lipophilic compounds accumulated in the cell membrane where the variation in chemical structure and thickness of carotenoids may affect the regulations of these compounds [54]. They present significant biological properties, for example, acting as antioxidants and protecting cells and tissues from the harmful effects of free radicals and singlet oxygen [55]. In addition, these compounds were reported to engage in photoprotection, and membrane stabilization [56,57,58]. Certain studies report that carotenoids possibly lowered the phase transition temperature of synthetic lipids and worked as regulators for membrane fluidity since they showed an important role in the causative function in the modulation of membrane parameters [59,60]. It was found that carotenoids increased membrane order with concurrent maintenance of lateral lipid motility, which resulted in a liquid-ordered membrane state [61] The increase in membrane order was indicated to be related to a simultaneous increase in membrane fluidity, and an increase in the concentration of carotenoids led to an increase in the resistance of the cell against freeze-thaw stress [54]. Furthermore, carotenoid biosynthesis can provide the ability of bacterial strains in destroying nonoxidative host defenses mediated by cationic peptides, by raising target membrane rigidity [62].

In the global cheese market, several cheese varieties have different shades of color, principally white or cream, pale yellow to intense yellow, and light orange to deep orange. The typical colors of cheese vary depending on the cheese type. Frequently, the development of pink color was discovered in a wide range of cheese categories and indicated as a defect. Pink discoloration generally declines consumer adoption since it is unusual for the color characteristics of cheeses. Physiochemical and biochemical factors can cause this coloration depending on the type of cheese, both with and without additional colorants. In terms of cheese without added colorant, pink coloration is mostly associated with microbial strains and their ability to produce red pigments in such a complex microbial community [63]. It has been reported that some strains of starter cultures applied for cheese production—e.g., *Lactobacillus* spp. and *Propionibacteria*, are involved in the pink coloration in cheese, as well as the activity of residual microbial enzymes [64,65]. Pink or red-pigmented species of the genus *Arthrobacter* were previously isolated from cheese; however, only a small number of their pigments have already been identified. *Arthrobacter* spp. including *A. aurescens*, *A. nicotianae*, and *A. globiformis* have been reported to synthesize red pigments during cheese production. Following absorption spectra measurements and chromatographic behavior of the red extracellular pigment in cultures, coproporphyrin III has been identified as the type of porphyrin produced by these bacteria [7,66]. Recently, the red pigments synthesized by *A. arilaitensis* strains grown on cheese-based (curd) solid medium deacidified using *D. hansenii* were identified. Based on the UV–Vis absorption spectra, the elution order, and fluorescent properties, compared to those of the porphyrin standards, eight metal-free porphyrins, including UPI, UPIII, 7PI, 6PI, 5PI, CPI, CPIII, and MPIX, were indicated as components of the red pigments extracted from a dry matter of the medium inoculated with *A. arilaitensis* strains [67]. In addition to well-known yellow-pigmented carotenoids, the genus *Arthrobacter* exhibits an ability to produce red carotenoids. Two species of this genus, *A. agilis* and *A. bussei*, have been reported for their involvement in the pink coloration in cheese, and the red pigments synthesized by them have been characterized as C_50_ carotenoid bacterirubirin and its glycosylated derivatives [49].

Despite available information regarding the diversity of bacteria belonging to the *Arthrobacter* spp. isolated from a variety of smear cheeses, very little is known about the coloration of cheese by these bacteria. Hereupon, for a purpose of providing information to cheese manufacturers when applying *Arthrobacter* spp. as a part of ripening flora, different aspects related to the cheese coloration should be investigated, e.g., the variation in pigment production among strains and factors affecting the pigmentation including physical and chemical factors, as well as the microbial interactions between *Arthrobacter* spp. and other microorganisms found on cheeses.

#### 3.1.2. Implication of *Arthrobacter* spp. in Cheese Flavor

Most flavor development by microorganisms occurs during the cheese-ripening process. The biochemical changes among diverse complex microbial communities involve the conversion of milk fat, carbohydrate, and protein to a very wide range of flavor compounds by three principal metabolic pathways, lipolysis, glycolysis, and proteolysis [68]. These pathways require several different enzymes that can be obtained from milk endogenous enzymes, clotting enzymes, and ripening microbial enzymes [69]. Cheeses produced from raw milk generally exhibit a more complex microbiota than cheeses made of pasteurized milk; as a consequence, a large quantity of aromatic compounds has been found in raw milk cheeses [70]. The degradation of amino acid by microbial enzymes, principally deaminase, transaminase, decarboxylase, lyase, and dehydratase, yields aldehydes, amines, acids, alcohols, and sulfur compounds; while the breakdown of fatty acid will deliver methyl ketones, secondary alcohols, and esters [71,72]. Depending on a variety of microflora presented during the ripening process, the substrates will be metabolized by several pathways to numerous compounds contributing to cheese flavor or off-flavors [68].

Among the microorganisms found on the surface of cheeses, a small number have been evaluated for their ability to produce flavor compounds, especially bacteria belonging to the genus *Arthrobacter*. Several strains of *A. arilaitensis* isolated from French smear-ripened cheeses were used to study the production of volatile aroma compounds, and it was found that various volatile compounds, e.g., aldehydes, esters, ketones, alcohols, and sulfur compounds, were produced by two strains, *A. arilaitensis* Mu107 and *A. arilaitensis* Po102 [69]. In a comparison study in lab-produced cheese between a typical old-young starter and a defined starter consisting of fungi and bacterial strains, a strain isolated from German Tilsit cheese, *A. nicotianae*, was the most important in the development of a typical smell—a sulfury volatile flavor [41]. The same study also reported that the sulfury volatile flavor formed by the defined starter on a pilot scale was very weak compared to the old-young smeared cheese. These results are consistent with a previous experiment that grew the strain in a liquid model system with only these two species [73]. 

A group of volatile sulfur compounds (VSCs) has been recognized as responsible for cheese flavor [74]. Most of these compounds are derivatives of the degradation of the sulfur-carbon bond of methionine to form methanethiol (MTL). The biosynthetic and catabolic pathways of methionine leading to MTL vary among microorganisms depending on their ability to synthesize methionine γ-lyase (MGL) [75]. In the cheese-ripening bacterium *B. linens*, two volatile sulfur compounds, MTL and α-ketobutyrate, are degraded-methionine products via this enzyme, and later MTL is converted to minor compounds including dimethyl disulfide (DMDS), dimethyl trisulfide (DMTS), and thioesters, while α-ketobutyrate is condensed with active acetaldehyde derived from pyruvate, and metabolized to 2,3-pentanedione (acetyl propionyl) [76]. In addition to *B. linens*, MGL is also considered to be present in several strains grown on the surface of cheeses such as *Micrococcus* spp., *Staphylococcus* spp., and *Arthrobacter* spp. [77]. A review of the *Arthrobacter* strain and its ability to produce numerous enzymes have revealed the potential of this genus for the synthesis of MGL [78,79]. 

**Figure 1:**
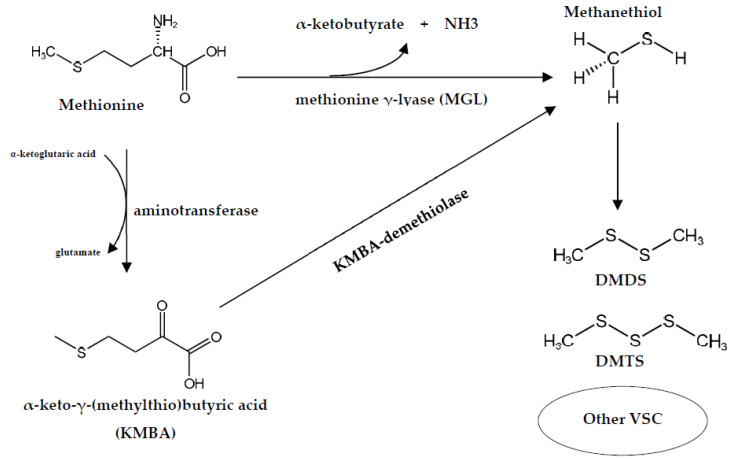
Methionine degradation pathways related to flavor formation in cheese by bacteria belonging to the genus *Arthrobacter*.

The production of MTL is generally associated with the one-step degradation of methionine by MGL; however, it can also originate in a two-step degradation pathway through two other enzymes, aminotransferase and α-keto-γ-(methylthio)-butyric acid demethiolase [80]. A study on the enzymatic pathways of methionine degradation to methanethiol in *Actinomycetes* bacteria isolated from ripened cheese found that the *Arthrobacter* sp. strain produced MTL, DMDS, and DMTS from both methionine and α-keto-γ-(methylthio)-butyric acid (KMBA) by enzymatic conversion through one-step and two-step degradation pathways, respectively [77]. The possibility of the enzymatic degradation of methionine via both pathways is shown in Figure 1 In addition, the ability of smear cheese-ripening bacteria to produce VSC from methionine is possibly influenced by cysteine as it appears likely that part of methionine can be converted into cysteine by the transsulfuration pathway through two major enzymes, i.e., cystathionine γ-synthase (CGS) and cystathionine β-lyase (CBL) [81]. CGL has been identified in bacteria as being responsible for hydrogen sulfide (H_2_S) while CBL releases homocysteine from cystathionine, which can either be involved in the biosynthesis of methionine or hydrolyzed by the CGL resulting in the production of H_2_S. Consequently, these two enzymes could reduce the amount of methionine, which would reduce VSC production [82]. The reduction of methionine catabolism by cysteine in *Arthrobacter* spp. could be caused by a reduction in MGL activity due either to a direct effect of high concentrations of cysteine or to other enzymes [74] 

Nowadays, many *Arthrobacter* strains isolated from a variety of cheeses have been discovered. Not only the yellow-pigmented *Arthrobacter* spp. but a large proportion of grey-white or cream-colored *Arthrobacter* isolates were also found on the surface of cheeses [41,83,84]. These pale-colored isolates are assumed to be responsible not for the cheese coloration but for the development of flavor and probably play a major role in cheese flavor formation. To achieve a better knowledge of the evolution of cheese flavor, the investigation mentioned that bacteria of the genus *Arthrobacter* may be required. The utilization in the cheese industry of bacteria belonging to this genus with specific flavor-forming abilities will be later considered as a promising tool to respond to the developing and controlling quality for organoleptic properties of cheese products.

### 3.2. Association of Arthrobacter spp. Producing Enzymes with Milk and Dairy Products

In the dairy industry, enzymes are widely used for the production and improvement of product quality, as well as for the innovation of new products. The industrial demand for new enzyme sources with different characteristics and low-cost production increases the number of studies related to the isolation and selection of new microorganisms. Among a diversity of microorganisms, psychrophilic bacteria are currently being intensively investigated due to their synthesis of cold-active enzymes. With a high possibility to be applied at low temperatures, especially in the food industry, such as in the processing of milk and dairy products, these enzymes are gaining commercial interest. Although several cold-active enzymes have been discovered, β-galactosidase, commonly known as lactase, which hydrolyzes lactose to glucose and galactose, seems to be a significant food-industrial enzyme because of its potential to degrade lactose for several purposes [85,86].

The principal industrial application of β-galactosidase is in the production of lactose-free milk and other related products—e.g., cheeses, yogurt, butter, and ice cream, for people who are lactose intolerant [87,88]. Generally, enzymatic lactose hydrolysis in milk is conducted under refrigerated conditions for approximately 24 h using commercial β-d-galactosidase extracted from mesophilic yeast *Kluyveromyces* spp., mainly *Kluyveromyces lactis*. The major disadvantage of the application of this enzyme in the dairy industry is its low activity under process conditions since it is optimally active at above 30 °C depending on the species of *Kluyveromyces* used as an enzyme producer [89,90]. Another significant application of β-d-galactosidase is the synthesis of galacto-oligosaccharides (GOS) from lactose. GOS are non-digestible oligosaccharides comprising 2 to 20 molecules of galactose and 1 molecule of glucose [91]. Recent studies ascribed several health benefits to these oligosaccharides [92]. GOS display functional prebiotic properties due to their stimulation of the proliferation of intestinal lactic acid bacteria and bifidobacteria [88]. GOS stimulates *Bifidobacterium* proliferation in the colon, which suppresses the activity of decomposing bacteria by antagonistic effect and diminishes the formation of toxic metabolites [93,94]. GOS are commercially available as prebiotics, with a low calorific value, and are reported to be capable of promoting satiety and reducing food intake [95].

The production of GOS from lactose through microbial β-galactosidases has been achieved using various enzymes deriving from mesophiles, thermophiles, and hyperthermophiles. By contrast, a small number of syntheses of GOS by the cold-active β-galactosidases from psychrophilic and psychrotolerant microorganisms have been studied [96]. Bioprospecting of microorganisms living in different cold environments led to the identification of cold-active β-galactosidases from many microbial species such as *Guehomyces*, *Thalassospira*, *Erwinia* [97,98,99].

Psychrophiles and psychrotolerant bacteria belonging to the genus *Arthrobacter*, especially strains isolated from extremely cold environments, have shown an ability to produce β-galactosidase [100]. Several Antarctic *Arthrobacter* strains, e.g., *Arthrobacter* sp. 20B, *Arthrobacter* sp. 32cB, *Arthrobacter* sp. ON14 and *Arthrobacter* sp. C2-2, *Arthrobacter psychrolactophilus* strain F2, and other unclassified *Arthrobacter* spp. were found to produce β-galactosidases, which were active at an optimum temperature range of 0–50 °C [88,101,102,103,104]. As a result of these discoveries, cold-active β-galactosidases produced by *A. psychrolactophilus* strain F2 are likely suitable enzymes to use under low-temperature conditions due to their activity being highest at 0 °C while their optimum temperature and pH were 10 °C and 8.0, respectively [105].

In addition to GOS, based on the ability of β-galactosidases to promote transglycosylation, isomerization, and transgalactosylation, this enzyme has been also used to produce hetero-oligosaccharides (HOS) by transferring the galactosyl moiety to sugars instead of lactose or its hydrolysis products, glucose, and galactose [87]. Lactulose, a well-known hetero-oligosaccharide, is a non-absorbable ketose disaccharide with prebiotic properties; therefore, like GOS, it is applied as a functional ingredient in the food industry and for pharmaceutical purposes [106]. Some strains of the genus *Arthrobacter* isolated from various diverse origins, such as *Arthrobacter* sp. from a fruit garden soil sample from China, *Arthrobacter* sp. 32cB from an Antarctic soil sample, and *Arthrobacter siccitolerans* sp. nov. from a dry soil sample from Spain, produce β-galactosidases which stimulates the hydrolysis of lactulose [106,107,108].

Technologically, lactose can easily be crystallized, which limits its applications in certain processes in the dairy industry. The application of β-galactosidases adds additional profitability to lactose-free or low-lactose dairy products as the hydrolysis of lactose will improve the solubility and digestibility of the dairy products [109]. It has been reported that using β-galactosidases to produce lactose-hydrolyzed yogurt can enhance lactic acid production and decrease whey separation as well as improve yogurt’s flavor, body, and texture [110]. In frozen dairy products, lactose hydrolysis by these enzymes improves the smoothness and scoop ability of the products due to the decrease in the freezing point [111]. The application of β-galactosidases in cheese production was found to reduce the ripening time and enrich the organoleptic properties of cheese. Therefore, this seems to be an effective element for reducing manufacturing costs as cheese ripening usually requires a long period of from six months to two years depending on the variety of cheeses [109,112]. In addition, β-galactosidases are considered a tool for adding value to by-products obtained from cheese production processes. Cheese whey is a by-product of the filtration process and it is recognized as a source of valuable molecules such as protein, lactose, and minerals; thus, it has been used as a substrate for the enzymatic production of GOS through the hydrolysis by β-galactosidases [113].

With the demand for appropriate and innovative technologies in the production of milk and milk-derived products as well as the by-product valorization, microbial β-galactosidases have been of increasing interest since they are very useful as catalysts that present several remarkable applications in the dairy industry. Based on the data obtained from previous studies about its thermostability, and thermoacidophilic and thermoresistant properties, the genus *Arthrobacter* promisingly provides advantages for use as a potential producer of β-galactosidases to fulfill commercial requirements as its enzymes allow dairy products to be treated under mild conditions.

### 3.3. Effect of Arthrobacter spp. on Nutritional Components in Milk and Dairy Products

Protein, fat, and carbohydrates, represent significant macronutrients in milk, accounting for 3–5, 4–6, and 4–5% of the total milk content, respectively [114]. Nevertheless, genetic, environmental, physiological, and handling variables are generally regarded as factors affecting milk composition. The contamination of *Arthrobacter* in milk and dairy products is possibly encountered either earlier in raw milk or later in processed products and can cause negative or positive effects on the quality of milk and its products in terms of their nutrition. According to genome sequencing studies, certain strains of bacteria in the genus *Arthrobacter* exhibit the genes encoding proteolytic, lipolytic, and glycolytic pathways [115,116,117]. These revelations confirm the results of several studies related to such enzyme-producing *Arthrobacter* bacteria [118,119]. Proteolysis, lipolysis, and glycolysis are normally considered the important elements of biochemical change in milk during fermentation leading to the nutritional value of final products.

#### 3.3.1. Proteolysis

Proteolysis is a hydrolysis reaction of peptide bonds in which proteins break down into smaller peptides and free amino acids. It is responsible for the metabolism of casein present in milk by the milk endogenous proteinase and other proteolytic enzymes of microorganisms present in milk; thus, leads to several biochemical reactions affecting nutritional and non-nutritional compounds—e.g., branched-chain and aromatic amino acids and sulfur-containing compounds. The branched-chain amino acids consist of leucine, isoleucine, and valine, whereas the aromatic amino acids include tryptophan, phenylalanine, and tyrosine [72]. The breakdown of amino acids via different pathways, namely decarboxylation and deamination, results in several compounds. Branched-amino acids can be decarboxylated using aminotransferases into amines, whereas dehydrogenases in deamination will convert these amino acids into carboxylic acids and ammonia [71]. Aromatic amino acids can also undergo decarboxylation and result in α-keto acids such as tryptophan to indole-3-pyruvate, tyrosine to hydroxybenzaldehyde, and phenylalanine to benzaldehyde [120].

Protein degradation generally occurs through extracellular enzymes synthesized by microorganisms during their growth or through intracellular enzymes produced up-on lysis. The synthesized proteinases expectedly involve casein degradation while those produced upon lysis tend to be peptidases and probably do not affect the degradation of casein but rather the breaking down of peptides originated by hydrolysis of proteinases [121]. By genome sequencing techniques, certain strains of *Arthrobacter* bacteria—e.g., *A. arilaitensis*, *A. aurescens*, and *A. chlorophenolicus*, demonstrate a number of genes encoding putative proteins with proteolytic functions [115,122].

The generation of peptides during microbial fermentation possibly produces the biologically active substances derived from milk protein. Milk-derived bioactive peptides are usually encrypted and inactive forms within the primary structure of milk protein and they are activated by proteolysis of casein namely α-, β-, γ- and κ-casein [123]. Numerous health-beneficial effects have been attributed to milk-derived bioactive peptides such as anti-hypertensive, hypolipidemic, anti-inflammatory, anti-oxidative, anti-microbial, and anti-osteoporotic effects [124,125,126]. In regards to health-promoting effects, milk-derived bioactive peptides are supposedly considered potential ingredients of functional foods.

#### 3.3.2. Lipolysis

Milk fat is primarily composed of triglycerides with phospholipids, cholesterol, and other lipids present at low concentrations. Milk triglycerides can be degraded through lipolytic pathways by endogenous enzymes in milk and microbial enzymes secreted during fermentation. The lipolytic enzymes can hydrolyze substrates to generate different forms of fatty acids, i.e., short- and intermediate-chain and free fatty acids [71]. The formation of these lipid molecules through lipolysis mostly reflects in the ester bonds between triglycerides and fatty acids cleaved by the lipases [127]. Fatty acids constitute a significant energy source for the growth of aerobic microorganisms. Some *Arthrobacter* including strains isolated from dairy products encode many proteins with putative lipase or esterase activity [115].

In the human body, free fatty acids (FFAs) serve in the first instance as an essential energy substrate and have a role in biological processes. FFAs can affect the gene expression of macrophages, adipocytes, or endothelial cells. In addition, FFAs can modulate the production of chemokines and cytokines and the expression of genes coding for adhesion molecules, and give rise to pro-inflammatory and inflammation-pro-resolving lipid-derived species [128]. High concentrations of FFAs are associated with some physiological conditions, for example, insulin resistance, fatty liver disease, atherosclerosis, and myocardial dysfunction [129]. Unlike FFAs, short-chain fatty acids (SCFAs) and medium-chain fatty acids (MCFAs) have many health benefits. The bioactivities of SCFAs, especially three major components, i.e., acetate, propionate, and butyrate, have been widely investigated. SCFAs demonstrate anti-inflammatory, immunoregulatory, anti-obesity, anti-diabetes, anticancer, cardiovascular protective, hepatoprotective, and neuroprotective effects [130]. For the regulation of MCFAs, these molecules modulate glucose and lipid metabolism. The unique transport and rapid metabolism of MCFAs provide additional health benefits compared to long-chain fatty acids (LCFAs); therefore, the use of MFAs for treating metabolic and neurological disorders has been of increasing interest [131].

#### 3.3.3. Glycolysis

Milk contains carbohydrates mainly in lactose form and trace amounts of monosaccharides and oligosaccharides. Lactose is a reducing disaccharide consisting of glucose and galactose. The metabolism of lactose to lactate is regularly metabolized by the glycolytic pathways, which are commonly associated with the fermentative metabolism in microorganisms. During fermentation, enzymes responsible for breaking down carbohydrates, which are synthesized by several bacteria contaminated in milk, hydrolyze lactose into its monomers, of which glucose will be further degraded. The principal products of lactose metabolism are L- or D-lactate, which are then oxidized into acetate and CO_2_ while some microbial strains create other products such as alcohol [132].

Cheese ripening is a type of milk fermentation that is probably most relevant to bacteria belonging to the genus *Arthrobacter*. Generally, the process of glycolysis in the degradation of milk carbohydrates to lactate seems to be responsible for lactic acid bacteria since they are the dominant bacterial species found at the beginning of the ripening process [72]. *Arthrobacter* strains that are frequently isolated from ripened cheeses may partly display a role in converting lactose to lactate due to their occurrence in the middle and late stages of the ripening process. Cheese-isolated *Arthrobacter* strains are aerobic respiratory bacteria and thus produced most of their ATP by oxidative phosphorylation. According to the presence of lactate at the beginning of ripening and quinone-dependent lactate dehydrogenases, glycolysis by ripened-cheese *Arthrobacter* is importantly related to the utilization of lactate as a carbon source [115]. However, several short-chain components—i.e., acetate, acetoin, diacetyl, ethanol, and acetaldehyde—are produced from an intermediate in lactose metabolism, pyruvate, as it is a precursor of these compounds [120]. The identification of gene encoding for enzymes required for the catabolism of carbohydrates sufficiently confirms the ability to utilize lactose as a substrate for glycolytic pathways [115,117].

Lactose-hydrolyzed milk (low-lactose and lactose-free milk) has been used for the preparation of flavored milk, cheese, yogurt, and other dairy products that serve as an important alternative for people who are lactose intolerant. The hydrolysis of lactose in milk for food processing also prevents lactose crystallization in frozen and condensed milk products; thus, it helps in improving the quality of the products [133]. Moreover, the use of hydrolyzed milk in yogurt and cheese manufacture accelerates the acidification process, because lactose hydrolysis is normally the rate-limiting step of the process, which reduces the set time of yogurt and accelerates the development of structure and flavor in cheese [134].

## 4. Conclusions

**Figure 2:**
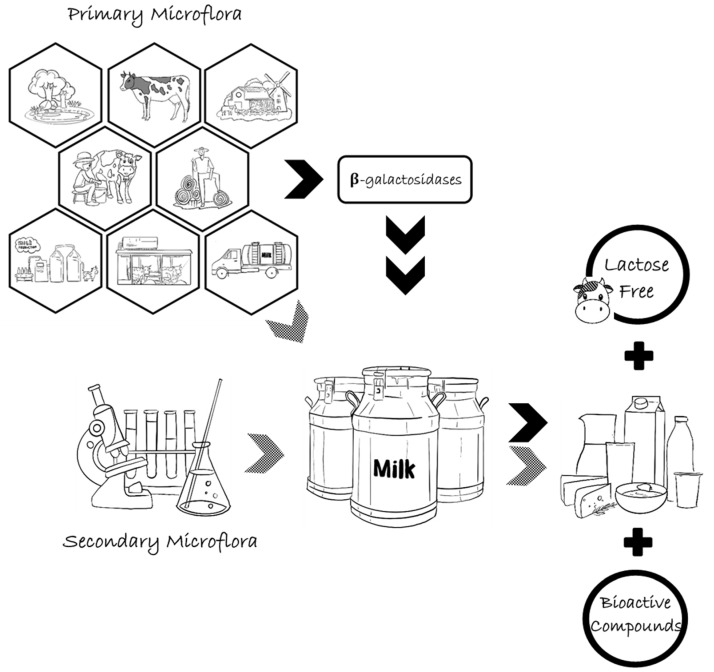
Potential origins of bacteria belonging to the genus *Arthrobacter* in milk and significant benefits of *Arthrobacter* spp. in the dairy industry.

Milk is a rich source of many essential nutrients, including protein, fats, carbohydrates, vitamins, minerals, and essential amino acids; as a result, it is quite a suitable medium for bacterial growth and can, therefore, serve as a source of bacterial contamination. Milk typically contains a variety of bacteria, of which some are beneficial such as those preserving milk through products of fermentation, e.g., cheese and yogurt. The genus *Arthrobacter* is among the major groups of microorganisms commonly found as a contaminant in milk and dairy products as well as being applied in the dairy industry. Almost all *Arthrobacter* spp. associated with fermented dairy products, especially a variety of cheeses, are considered microflora that enter milk or dairy products from the environment and milking equipment. They are mostly responsible for the coloration and flavor formation of cheeses, whilst a small number of *Arthrobacter* spp. isolated from extreme environments are employed as producers of β-galactosidase used for lactose hydrolysis in order to produce GOS and other lactose derivatives. Because of these beneficial aspects derived from bacteria belonging to the genus *Arthrobacter* including pigmentation, flavor formation, and enzyme production, the genus *Arthrobacter* is a promising bacterial genus for use in the production of products such as cheeses low-lactose and lactose-free dairy products, and functional dairy products (Figure 2). However, a better understanding of its roles related to these aspects is still required to enlarge the available benefits. Meanwhile, the isolation of new strains for use in the production of pigments, flavor compounds, and enzymes will possibly meet the demands of the dairy industry and beyond.

## Data Availability

Not applicable.
